# Taxonomy based on the biological species concept: the case of the *Rhodnius robustus *complex (Hemiptera, Triatominae)

**DOI:** 10.1186/s13071-025-07045-x

**Published:** 2025-10-21

**Authors:** Kaio Cesar Chaboli Alevi, Amanda Ravazi, Jader de Oliveira, Yago Visinho dos Reis, Isadora de Freitas Bittinelli, Luiza Maria Grzyb Delgado, Jociel Klleyton Santos Santana, Maria Tercília Vilela de Azeredo-Oliveira, João Aristeu da Rosa, Mauro Toledo Marrelli, Cleber Galvão

**Affiliations:** 1 Laboratory of Entomology in Public Health, Department of Epidemiology, Faculty of Public Health (FSP), University of Sao Paulo (USP), Avenue Dr. Arnaldo, 715, Sao Paulo, SP 01246-904 Brazil; 2 National and International Reference Laboratory On Triatomine Taxonomy, Oswaldo Cruz Institute (IOC), Oswaldo Cruz Foundation (FIOCRUZ), Avenue Brasil, 4365, Rio de Janeiro, RJ 21040-360 Brazil; 3 Institute of Biosciences, São Paulo State University (UNESP), Botucatu, 18618-689 Brazil; 4 Laboratory of Parasitology, School of Pharmaceutical Sciences (FCFAR), São Paulo State University (UNESP), road Araraquara/Jau, Km 01, Araraquara, SP 14801-902 Brazil; 5 Department of Entomology, National Museum of Natural History, Smithsonian Institution, Washington, D.C., USA; 6 Institute of Biosciences, Humanities and Exact Sciences (IBILCE), São Paulo State University (UNESP), São José do Rio Preto, 15054-000 Brazil

**Keywords:** Rhodniini, Experimental crosses, Prezygotic barriers, Hybrid inviability, Hybrid sterility, Hybrid colapse

## Abstract

**Background:**

*Rhodnius robustus* is a paraphyletic taxon composed of different lineages, being lineages II and III currently described as *R*. *montenegrensis* and *R*. *marabaensis*. However, it has been suggested that two distinct clades may be associated with lineage II: *R*. *montenegrensis* and *R*. *robustus* II. Among the different species concepts, the biological concept is based mainly on interspecific reproductive isolation. Thus, we performed crosses between *R*. *montenegrensis* and *R*. *marabaensis* and between *R*. *montenegrensis* and *R*. *robustus* II, and we revisited and discussed taxonomically and evolutionarily all intra and interspecific crosses of *R*. *robustus*  *sensu* *lato (s. l.)*.

**Methods:**

Reciprocal experimental crosses were conducted between *R*. *montenegrensis* with *R*. *marabaensis* and *R*. *robustus* II. Furthermore, intercrosses were performed up to the fourth (F4) or fifth generation (depending on the reproductive barrier). Finally, cytogenetic analyzes were performed in the gonads to evaluate the degree of pairing of the hybrids’ chromosomes.

**Results:**

Hybrids were obtained in all directions of the crosses. However, crosses between *R*. *montenegrensis* females and *R*. *marabaensis* males resulted in a very low number of offspring. When intercrosses were performed, the hybrids showed high fertility, possibly resulting from heterosis. Only the F4 was unviable due to hybrid collapse. In the other direction, the hybrids were completely infertile, characterizing the hybrid sterility. Hybrids from crosses between *R*. *montenegrensis* females and *R*. *robustus* II males died before reaching adulthood (characterizing the hybrid collapse) and in the other direction, no reproductive barriers were observed.

**Conclusions:**

We demonstrated that, on the basis of the biological species concept, *R*. *montenegrensis* and *R*. *marabaensis* are valid species and reproductively isolated. Furthermore, we observed partial isolation between *R*. *montenegrensis* and *R*. *robustus* II, highlighting the need for integrative taxonomy studies to elucidate this clade. Finally, we revisited the literature and observed that: *R*. *robustus s*. *l*. is reproductively isolated from *R*. *nasutus* and *R*. *neglectus*; with the exception of specimens from Amazonas, *R*. *prolixus* does not present reproductive isolation when crossed with *R*. *robustus* (from Colombia, Peru and Venezuela); and the “intraspecific” barriers of *R*. *robustus s*. *l*. possibly represent different species (*R*. *montenegrensis*, *R*. *marabaensis*, and/or *R*. *barretti*).

**Graphical Abstract:**

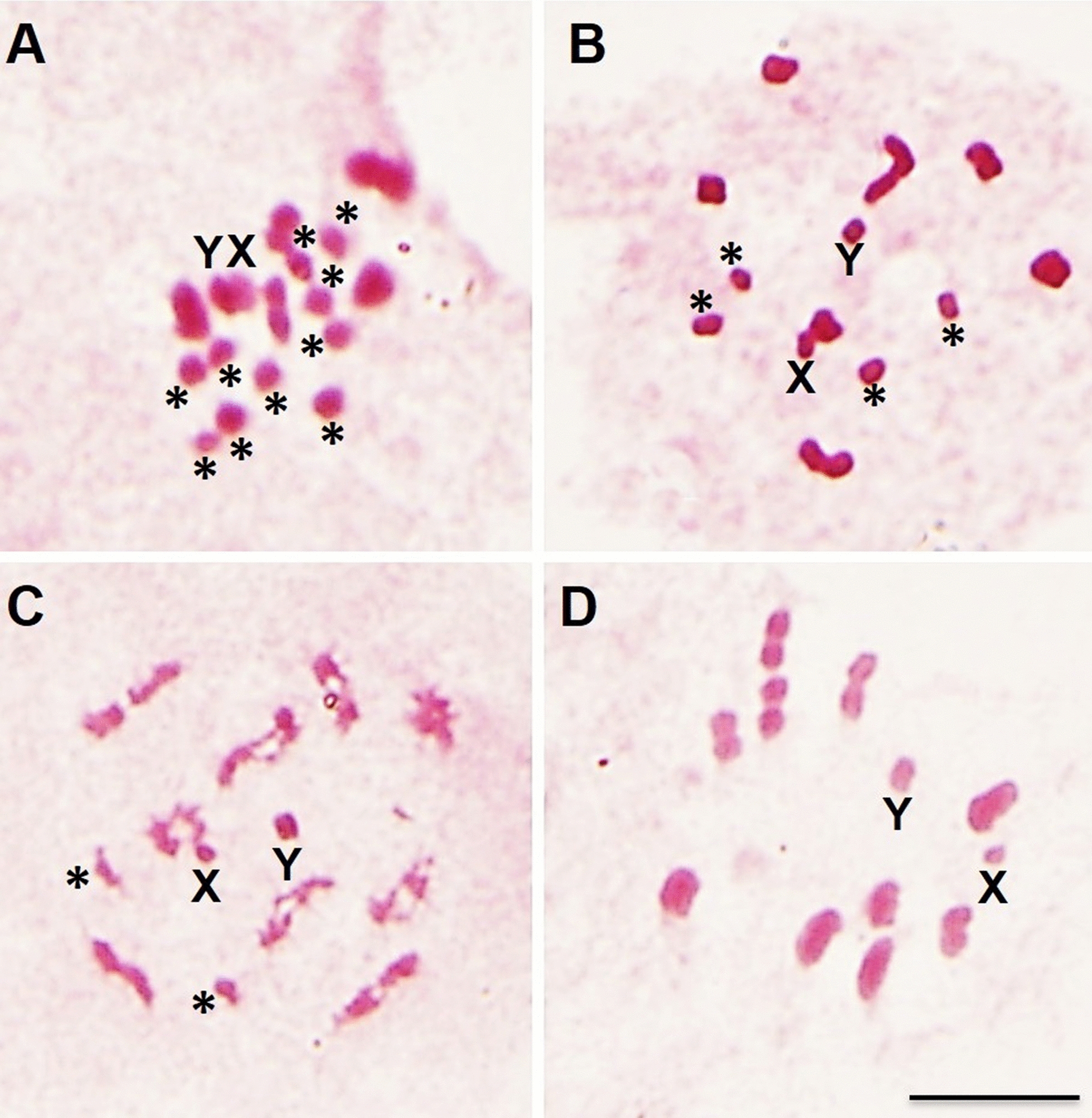

## Background

Almost a century after the description of *Rhodnius robustus* Larrousse, 1927 [1], the species continues to be the subject of taxonomic and evolutionary discussions [2]. The taxonomic problem began in the description article itself, once Larrousse [1] characterized the species on the basis of two females from different localities (one from the region of Cayenne, in French Guiana, and the other from the mouth of the Tefé river, in the Brazilian Amazon) and did not indicate which was the holotype. On the other hand, the evolutionary problem was highlighted in 2003, when Monteiro et al. [3], through phylogenetic studies, demonstrated that *R*. *robustus* did not represent a single taxon, but rather a paraphyletic species complex with four different lineages (I from the Orinoco and, II, III, and IV, from the Amazon region—named here as *R*. *robustus* I-IV).

Currently, at least five lineages that derived from a late Pliocene–Pleistocene radiation are recognized, namely, a northern cluster from the Orinoco basin (*R*. *robustus* I), a core-Amazon cluster, including *R*. *robustus* II, *R*. *robustus* III, and *R*. *robustus* IV (occupying the northeastern Amazon including the Guiana highlands), as well as, a cluster from the central-northern Amazon (*R*. *robustus* V) [4]. The characterization of these different evolutionary lineages (which represented different taxa) demonstrated that the two syntype females used by Larrousse [1] were possibly two genetically different lineages/species, but morphologically indistinguishable (cryptic) [3]. This fact makes it even more difficult to use of the *R*. *robustus* syntypes [1] in taxonomic studies, being essential the designation of a lectotype among them.

*Rhodnius robustus* was described as a sister taxon of *R*. *prolixus* Stål, 1859 [1]. Brito et al. [5] highlighted that *R*. *prolixus* and *R*. *robustus sensu* *lato* (*s. l.*) are similar in many aspects, but differ epidemiologically: the former is a primary domestic vector of Chagas disease, whereas the latter comprises a suite of sylvatic species of limited medical relevance. Although Larrousse [1] used phenotypic differences to differentiate the species, overlapping diagnostic characters were observed [6, 7]—which makes it difficult to differentiate these taxa only by classical taxonomy, [6, 7]— resulting in the questioning of the specific status of *R*. *robustus* [7]. Thus, several comparative studies were carried out between *R*. *robustus s. l.* and *R*. *prolixus*, to assist in the taxonomy of the species [8–14].

Furthermore, studies related with each lineage of *R*. *robustus sensu* *stricto* (*s. s.*) were also carried out and, through integrative taxonomy studies, some lineages were described as new species: *R*. *robustus* II as *R*. *montenegrensis* Rosa et al. [15] and *R*. *robustus* III, as *R*. *marabaensis* Souza et al. [16]. Despite efforts to differentiate *R*. *montenegrensis* and *R*. *robustus* II [15, 17], Brito et al. [5] emphasized that they represented the same lineage (which does not invalidate the specific status of *R*. *montenegrensis*, since a paraphyletic taxon does not satisfy any of the existing species concepts [18–20]).

A recent phylogenomic study reconstructed the evolutionary history of the Rhodniini tribe and, among the various taxonomic and evolutionary inferences, was suggested that *R*. *montenegrensis* is a valid taxon that represents only a part of the specimens of lineage II [2]. On the contrary, Filée et al. [2] confirmed the specific status of *R*. *marabaensis* and highlighted that this taxon really represented lineage III (as initially suggested by Monteiro et al. [4]). Finally, the authors suggest that *R*. *montenegrensis*, *R*. *marabaensis*, *R*. *robustus* and *R*. *prolixus* should be grouped into the *R*. *robustus* complex [2].

Among the different species concepts, the biological concept is based mainly on interspecific reproductive isolation [21–24]. Curiously, several attempts at crossing *R*. *robustus s. l.* and *R*. *prolixus* were viable and did not present reproductive barriers (mainly in the direction of *R*. *robustus s. l.* females and *R*. *prolixus* males) [25–27], while many “intraspecific” crosses between *R*. *robustus s. l.* allowed the observation of postzygotic barriers [25, 26]. Under laboratory conditions, the absence of reproductive barriers does not allow taxonomic changes to be proposed (since some natural prezygotic barriers are not considered [21–25]), but, when pre or postzygotic barriers are characterized (even in just one direction), the specific status of the parents can be confirmed or, above all, questioned (confirming that the lineages represent different taxa).

Thus, we performed crosses between *R*. *montenegrensis* and *R*. *marabaensis* and between *R*. *montenegrensis* and *R*. *robustus* II, to evaluate the possible reproductive barriers established between these species of the *R*. *robustus* complex. In addition, we revisited and discussed taxonomically and evolutionarily all intra and interspecific crosses of *R. robustus s. l*.

## Methods

### Experimental crosses

Reciprocal experimental crosses were conducted between *R*. *montenegrensis* (from Rondônia, Brazil—colony started in 2008, with approximately 51 generations) [28] with *R*. *marabaensis* (from Pará, Brazil—colony started in 2014, with approximately 33 generations) [29] and *R*. *robustus* II (from Lima, Peru—colony started in 1972, with approximately 159 generations) [30] (Table 1). The insects used in these experiments were obtained from colonies kept in the Triatominae insectary of the School of Pharmaceutical Sciences, São Paulo State University (FCFAR/UNESP), Araraquara, São Paulo, Brazil. Species identification was conducted with the help of the dichotomous keys developed by Galvão al. [31], as well as from the diagnostic characteristics of each species [1, 15, 16]. The experimental crosses were conducted in the Triatominae insectary of FCFAR/UNESP, according to the experiments of Mendonça et al. [32] and Reis et al. [33]: fifth-instar nymphs (N5) were sexed and males and females were kept separately until they reached the adult stage to guarantee the virginity of the insects used in the crosses. For the experimental crosses, three couples from each set were placed separately in plastic jars (diameter 5 cm × height 10 cm) and kept at room temperature (average of 24 ℃) and relative humidity of 63% [34]. Furthermore, intraspecific crosses were also performed for group control (Table 1). The eggs were collected weekly throughout the female’s oviposition periods and the egg fertility rate was calculated (Table 1). Additionally, after the hybrids hatched, the development of first instar-nymphs until adults was also monitored weekly to assess the mortality rate (Table 1). With the first-generation hybrids (F1) nymphs that have reached adulthood, six new couples of F1 hybrids (three for each direction) were separated for intercrossing, with the same parameters described used in the evaluation (Table 1). Furthermore, intercrosses between second-generation hybrids (F2) were also carried out in both directions (Table 1). This was repeated up to the fourth-generation hybrids (F4) for the crosses between *R*. *montenegrensis* females and *R*. *marabaensis* males, and between *R*. *montenegrensis* females and *R*. *robustus* males, and up to the fifth-generation (F5) for the cross between *R*. *robustus* females and *R*. *montenegrensis* males (Table 1). We justify that for all quantitative data collected, the relative frequency was calculated.

**Table 1:** Experimental crosses performed between *R*. *montenegrensis* with *R*. *marabaensis*, and *R*. *robustus* II

	Experimental crosses		Number of eggs				Egg fertility
	Interspecific crosses		C1	C2	C3	Total	
♀	*R*. *marabaensis* × *R*. *montenegrensis*^1^	♂	97	54	62	213	128 (60%)
♀	*R*. *montenegrensis* × *R*. *marabaensis*^2^	♂	121	123	192	436	36 (08%)
♀	*R*. *montenegrensis* × *R*. *robustus*^3^	♂	147	208	210	565	331 (59%)
♀	*R*. *robustus* × *R*. *montenegrensis*^4^	♂	241	309	302	852	484 (57%)
	Intercrosses						
♀	Hybrid F1^1^ × Hybrid F1^1^	♂	73	76	62	211	00 (00%)
♀	Hybrid F1^2^ × Hybrid F1^2^	♂	22	25	13	60	42 (70%)
♀	Hybrid F2^2^ × Hybrid F2^2^	♂	111	228	274	171	42 (25%)
♀	Hybrid F3^2^ × Hybrid F3^2^	♂	77	101	98	107	49 (46%)
♀	Hybrid F4^2^ × Hybrid F4^2^	♂	–	–	–	–	00 (00%)^+^
♀	Hybrid F1^3^ × Hybrid F1^3^	♂	136	279	112	316	69 (22%)
♀	Hybrid F2^3^ × Hybrid F2^3^	♂	56	224	67	347	279 (80%)
♀	Hybrid F3^3^ × Hybrid F3^3^	♂	20	00	81	101	27 (27%)
♀	Hybrid F4^3^ × Hybrid F4^3^	♂	11	07	00	18	02 (11%)*
♀	Hybrid F1^4^ × Hybrid F1^4^	♂	295	356	263	887	681 (77%)
♀	Hybrid F2^4^ × Hybrid F2^4^	♂	14	59	130	203	89 (44%)
♀	Hybrid F3^4^ × Hybrid F3^4^	♂	67	10	126	203	69 (30%)
♀	Hybrid F4^4^ × Hybrid F4^4^	♂	72	86	64	222	149 (67%)
♀	Hybrid F5^4^ × Hybrid F5^4^	♂	110	100	117	327	90 (28%)
	Control experiments						
♀	*R*. *marabaensis* × *R*. *marabaensis*	♂	63	46	–	109	75 (69%)
♀	*R*. *montenegrensis* × *R*. *montenegrensis*	♂	131	249	–	380	266 (70%)
♀	*R*. *robustus* × *R*. *robustus*	♂	173	194	–	367	269 (73%)

^+^All adults died before oviposition

^*^All offspring died before reaching adulthood

### Cytological analysis

Five adult male hybrids from each generation (F1-F5) were dissected and their testes were removed and stored in a methanol: acetic acid solution (3:1). Slides were prepared by the cell-crushing technique (as described by Alevi et al. [35]), and cytogenetic analyses were performed to characterize spermatogenesis, with emphasis on the degree of pairing between the homologous chromosomes, using the lacto-acetic orcein technique [35, 36]. The slides were examined under a light microscope (Jenamed; Carl Zeiss, Jena, Germany) that was coupled with a digital camera with a 1000-fold magnification; AxioVision LE version 4.8 imaging software (Carl Zeiss) was used for analysis.

## Results

**Fig. 1:**
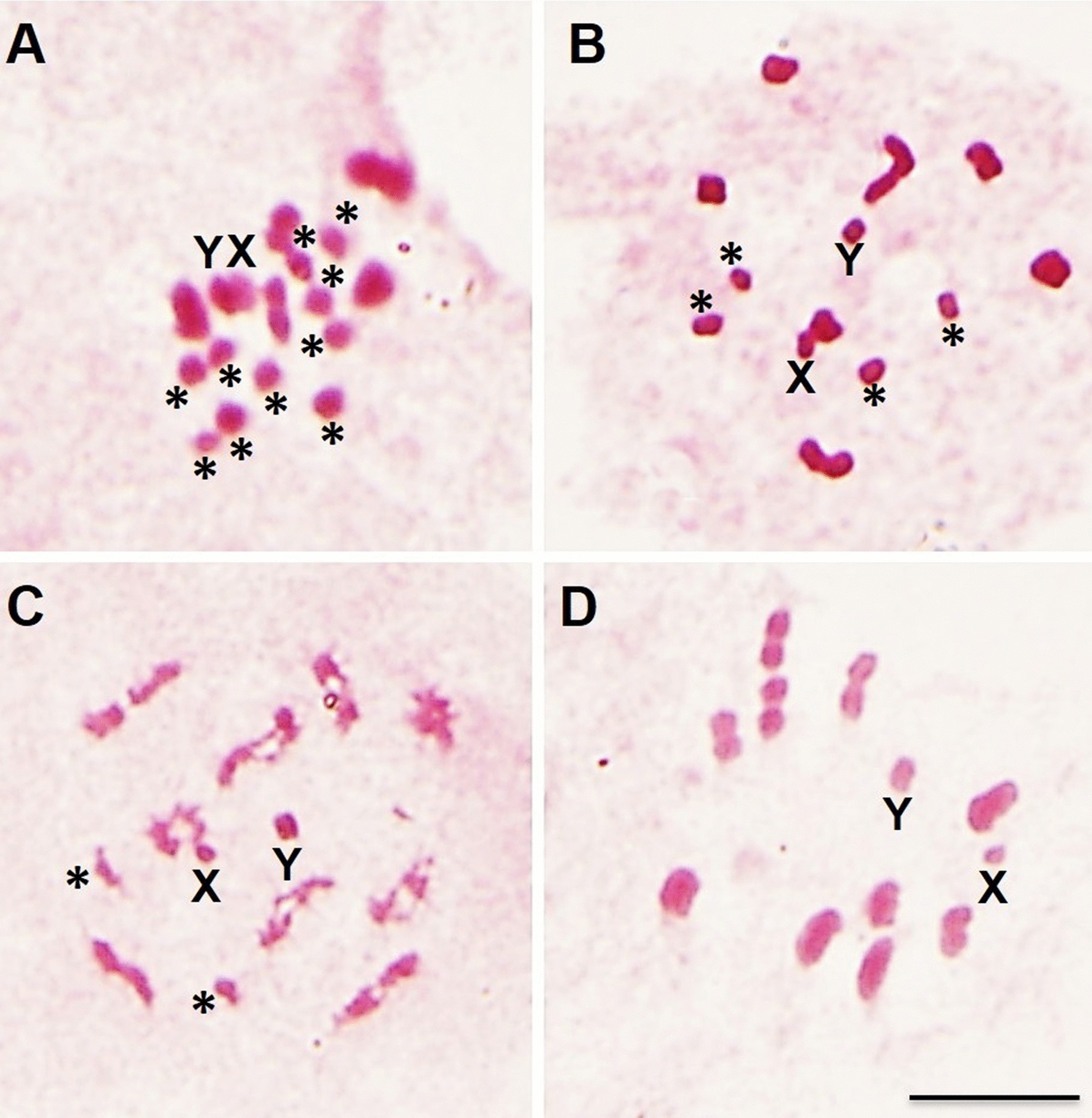
Prometaphases and metaphases of hybrids from: the cross between *R*. *montenegrensis* ♀ × *R*. *marabaensis* ♂ of fourth generation (**A**); the cross between *R*. *montenegrensis* ♂ × *R*. *marabaensis* ♀ of first generation (**B**); the cross between *R*. *montenegrensis* ♀ × *R*. *robustus* II ♂ of fourth generation (**C**); and from the cross between *R*. *robustus* II ♀ × *R*. *montenegrensis* ♂ of fifth generation (**D**). **A, B, C**: Note pairing errors between different autosomes (asterisk). **D**: Note that 100% of the chromosomes were paired. X: sex chromosome; Y: Y sex chromosome; Bar: 10 µm

Hybrids were obtained in all directions of the crosses (Table 1). However, crosses between *R*. *montenegrensis* females and *R*. *marabaensis* males resulted in a very low number of offspring, but when intercrosses were performed, the hybrids showed high fertility (Table 1). Only F4 was unviable owing to hybrid collapse (Tables 1 and 2)—with chromosome pairing errors (Fig. 1A). In the other direction, the hybrids were completely infertile, characterizing the hybrid sterility (Tables 1 and 2)—also with chromosome pairing errors (Fig. 1B). F4 hybrids from crosses between *R*. *montenegrensis* females and *R*. *robustus* II males died before reaching adulthood, characterizing the hybrid collapse (Tables 1 and 2). Cytogenetic analysis of a N5 male nymphs demonstrated that these organisms presented pairing errors in one pair of autosomes (Fig. 1C). In the other direction, reproductive barriers (Tables 1 and 2) and chromosome pairing errors (Fig. 1D) were not observed.

**Table 2:** Intra and interspecific experimental crosses performed with *R*. *robustus s. l*

Experimental crosses	Prezygotic barrier	Poszygotic barrier			References
Interspecific crosses		Hybrid inviability	Hybrid sterility	Hybrid collapse	
*R*. *marabaensis* (Pará, Brazil) ♀ × *R*. *montenegrensis* (Rondônia, Brazil) ♂	Absent	–	Present	–	This paper
*R*. *montenegrensis* (Rondônia, Brazil) ♀ × *R*. *marabaensis* (Pará, Brazil) ♂	Absent	–	–	Present	This paper
*R*. *montenegrensis* (Rondônia, Brazil) ♀ × *R*. *robustus* (Lima, Peru) ♂	Absent	–	–	Present	This paper
*R*. *robustus* (Lima, Peru) ♀ × *R*. *montenegrensis* (Rondônia, Brazil) ♂	Absent	Absent	Absent	Absent	This paper
*R*. *robustus* (Santander, Colombia) ♀ × *R*. *nasutus* (Ceará, Brazil) ♂	Absent	–	Present (♂)^2^	Present^1^	[25, 26]
*R*. *robustus* (Lima, Peru) ♀ × *R*. *nasutus* (Ceará, Brazil) ♂	Absent	–	Present	–	[25, 26]
*R*. *robustus* (Pará, Brazil) ♀ × *R*. *nasutus* (Ceará, Brazil) ♂	Absent	–	Present (♂)^2^	–	[25, 26]
*R*. *robustus* (Rondônia, Brazil) ♀ × *R*. *nasutus* (Ceará, Brazil) ♂	Absent	–	Present (♂)^2^	Present^1^	[25, 26]
*R*. *prolixus*/*R*. robustus* (Boyaca, Colombia) ♀ × *R*. *nasutus* (Ceará, Brazil) ♂	Present	–	–	–	[25, 26]
*R*. *nasutus* (Ceará, Brazil) ♀ × *R*. *robustus* (Santander, Colombia) ♂	Present	–	–	–	[25, 26]
*R*. *nasutus* (Ceará, Brazil) ♀ × *R*. *robustus* (Lima, Peru) ♂	Present	–	–	–	[25, 26]
*R*. *nasutus* (Ceará, Brazil) ♀ × *R*. *prolixus*/*R*. robustus* (Boyaca, Colombia) ♂	Present	–	–	–	[25, 26]
*R*. *nasutus* (Ceará, Brazil) ♀ × *R*. *robustus* (Pará, Brazil) ♂	Absent	–	Present^1^	–	[25, 26]
*R*. *nasutus* (Ceará, Brazil) ♀ × *R*. *robustus* (Rondônia, Brazil) ♂	Absent	–	Present^1^	–	[25, 26]
*R*. *robustus* (Santander, Colombia) ♀ × *R*. *neglectus* (Bahia, Brazil) ♂	Present	–	–	–	[25, 26]
*R*. *robustus* (Santander, Colombia) ♀ × *R*. *neglectus* (São Paulo, Brazil) ♂	Present	–	–	–	[25, 26]
*R*. *robustus* (Santander, Colombia) ♀ × *R*. *neglectus* (Tocantins, Brazil) ♂	Absent	–	Present (♂)^2^	–	[25, 26]
*R*. *robustus* (Santander, Colombia) ♀ × *R*. *neglectus* (Goiás, Brazil) ♂	Absent	–	Present (♂)^2^	–	[25, 26]
*R*. *robustus* (Lima, Peru) ♀ × *R*. *neglectus* (Bahia, Brazil) ♂	Present	–	–	–	[25, 26]
*R*. *robustus* (Lima, Peru) ♀ × *R*. *neglectus* (São Paulo, Brazil) ♂	Present	–	–	–	[25, 26]
*R*. *robustus* (Lima, Peru) ♀ × *R*. *neglectus* (Tocantins, Brazil) ♂	Present	–	–	–	[25, 26]
*R*. *robustus* (Lima, Peru) ♀ × *R*. *neglectus* (Goiás, Brazil) ♂	Present	–	–	–	[25, 26]
*R*. *robustus* (Rondônia, Brazil) ♀ × *R*. *neglectus* (Goiás, Brazil) ♂	Absent	–	Present (♂)^2^	–	[25, 26]
*R*. *robustus* (Rondônia, Brazil) ♀ × *R*. *neglectus* (Tocantins, Brazil) ♂	Absent	–	Present (♂)^2^	–	[25, 26]
*R*. *robustus* (Pará, Brazil) ♀ × *R*. *neglectus* (Tocantins, Brazil) ♂	Absent	–	Present (♂)^2^	Present^1^	[25, 26]
*R*. *robustus* (Pará, Brazil) ♀ × *R*. *neglectus* (Goiás, Brazil) ♂	Absent	–	Present (♂)^2^	Present^1^	[25, 26]
*R*. *robustus* (Pará, Brazil) ♀ × *R*. *neglectus* (Bahia, Brazil) ♂	Absent	–	Present (♂)^2^	–	[25, 26]
*R*. *robustus* (Pará, Brazil) ♀ × *R*. *neglectus* (São Paulo, Brazil) ♂	Absent	–	Present (♂)^2^	–	[25, 26]
*R*. *prolixus*/*R*. robustus* (Boyaca, Colombia) ♀ × *R*. *neglectus* (São Paulo, Brazil) ♂	Present	–	–	–	[25, 26]
*R*. *prolixus*/*R*. robustus* (Boyaca, Colombia) ♀ × *R*. *neglectus* (Bahia, Brazil) ♂	Present	–	–	–	[25, 26]
*R*. *prolixus*/*R*. robustus* (Boyaca, Colombia) ♀ × *R*. *neglectus* (Goiás, Brazil) ♂	Absent	–	Present (♂)^2^	–	[25, 26]
*R*. *prolixus*/*R*. robustus* (Boyaca, Colombia) ♀ × *R*. *neglectus* (Tocantins, Brazil) ♂	Absent	–	Present (♂)^2^	–	[25, 26]
*R*. *neglectus* (Tocantins, Brazil) ♀ × *R*. *robustus* (Santander, Colombia) ♂	Present	–	–	–	[25, 26]
*R*. *neglectus* (Tocantins, Brazil) ♀ × *R*. *robustus* (Lima, Peru) ♂	Present	–	–	–	[25, 26]
*R*. *neglectus* (Tocantins, Brazil) ♀ × *R*. *robustus* (Pará, Brazil) ♂	Absent	–	–	Present^1^	[25, 26]
*R*. *neglectus* (Goiás, Brazil) ♀ × *R*. *robustus* (Pará, Brazil) ♂	Absent	–	–	Present^1^	[25, 26]
*R*. *neglectus* (Goiás, Brazil) ♀ × *R*. *robustus* (Santander, Colombia) ♂	Present	–	–	–	[25, 26]
*R*. *neglectus* (Goiás, Brazil) ♀ × *R*. *robustus* (Lima, Peru) ♂	Present	–	–	–	[25, 26]
*R*. *neglectus* (Bahia, Brazil) ♀ × *R*. *robustus* (Pará, Brazil) ♂	Absent	–	–	Present^1^	[25, 26]
*R*. *neglectus* (Bahia, Brazil) ♀ × *R*. *robustus* (Santander, Colombia) ♂	Present	–	–	–	[25, 26]
*R*. *neglectus* (Bahia, Brazil) ♀ × *R*. *robustus* (Lima, Peru) ♂	Present	–	–	–	[25, 26]
*R*. *neglectus* (São Paulo, Brazil) ♀ × *R*. *robustus* (Santander, Colombia) ♂	Present	–	–	–	[25, 26]
*R*. *neglectus* (São Paulo, Brazil) ♀ × *R*. *robustus* (Lima, Peru) ♂	Present	–	–	–	[25, 26]
*R*. *neglectus* (São Paulo, Brazil) ♀ × *R*. *robustus* (Pará, Brazil) ♂	Absent	–	–	Present^1^	[25, 26]
*R*. *neglectus* (Bahia, Brazil) ♀ × *R*. *prolixus*/*R*. robustus* (Boyaca, Colombia) ♂	Present	–	–	–	[25, 26]
*R*. *neglectus* (São Paulo, Brazil) ♀ × *R*. *prolixus*/*R*. robustus* (Boyaca, Colombia) ♂	Present	–	–	–	[25, 26]
*R*. *neglectus* (Goiás, Brazil) ♀ × *R*. *prolixus*/*R*. robustus* (Boyaca, Colombia) ♂	Present	–	–	–	[25, 26]
*R*. *neglectus* (Tocantins, Brazil) ♀ × *R*. *prolixus*/*R*. robustus* (Boyaca, Colombia) ♂	Present	–	–	–	[25, 26]
*R*. *robustus* (Pará, Brazil) ♀ × *R*. *prolixus* (Amazonas, Brazil) ♂	Absent	–	Present (♀)^1, 2^	–	[25, 26]
*R*. *robustus* (Pará, Brazil) ♀ × *R*. *prolixus* (Cojedes, Venezuela) ♂	Absent	–	Present	–	[25, 26]
*R*. *robustus* (Pará, Brazil) ♀ × *R*. *prolixus* (Cundinamarca, Colombia) ♂	Absent	–	Present^1,3^	–	[25, 26]
*R*. *robustus* (Pará, Brazil) ♀ × *R*. *prolixus* (Honduras) ♂	Absent	–	Present (♂)^1,2^	–	[25, 26]
*R*. *robustus* (Pará, Brazil) ♀ × *R*. *prolixus* (Casanare, Colombia) ♂	Absent	–	Present^1^	–	[25, 26]
*R*. *robustus* (Rondônia, Brazil) ♀ × *R*. *prolixus* (Cojedes, Venezuela) ♂	Absent	–	–	–	[25, 26]
*R*. *robustus* (Rondônia, Brazil) ♀ × *R*. *prolixus* (Cundinamarca, Colombia) ♂	Absent	–	–	–	[25, 26]
*R*. *robustus* (Rondônia, Brazil) ♀ × *R*. *prolixus* (Honduras) ♂	Absent	Present	–	–	[25, 26]
*R*. *robustus* (Rondônia, Brazil) ♀ × *R*. *prolixus* (Casanare, Colombia) ♂	Absent	–	Present (♀)^1,2^	–	[25, 26]
*R*. *robustus* (Rondônia, Brazil) ♀ × *R*. *prolixus* (Amazonas, Brazil) ♂	Absent	Present^2^	–	–	[25, 26]
*R*. *robustus* (Santander, Colombia) ♀ × *R*. *prolixus* (Cojedes, Venezuela) ♂	Absent	Absent	Absent	Absent	[25, 26]
*R*. *robustus* (Santander, Colombia) ♀ × *R*. *prolixus* (Cundinamarca, Colombia) ♂	Absent	Absent	Absent	Absent	[25, 26]
*R*. *robustus* (Santander, Colombia) ♀ × *R*. *prolixus* (Honduras) ♂	Absent	Absent	Absent	Absent	[25, 26]
*R*. *robustus* (Santander, Colombia) ♀ × *R*. *prolixus* (Casanare, Colombia) ♂	Absent	Absent	Absent	Absent	[25, 26]
*R*. *robustus* (Santander, Colombia) ♀ × *R*. *prolixus* (Amazonas, Brazil) ♂	Absent	Present	Present (♂)^1,2^	–	[25, 26]
*R*. *robustus* (Lima, Peru) ♀ × *R*. *prolixus* (Cojedes, Venezuela) ♂	Absent	Absent	Absent	Absent	[25, 26]
*R*. *robustus* (Lima, Peru) ♀ × *R*. *prolixus* (Cundinamarca, Colombia) ♂	Absent	Absent	Absent	Absent	[25, 26]
*R*. *robustus* (Lima, Peru) ♀ × *R*. *prolixus* (Honduras) ♂	Absent	Absent	Absent	Absent	[25, 26]
*R*. *robustus* (Lima, Peru) ♀ × *R*. *prolixus* (Casanare, Colombia) ♂	Absent	Absent	Absent	Absent	[25, 26]
*R*. *robustus* (Lima, Peru) ♀ × *R*. *prolixus* (Amazonas, Brazil) ♂	Absent	Present	Present (♂)^1,2^	–	[25, 26]
*R*. *robustus* (Mérida, Venezuela) ♀ × *R*. *prolixus* (Lara, Venezuela) ♂	Absent	Absent	Absent	Absent	[27]
*R*. *robustus* (Mérida, Venezuela) ♀ × *R*. *prolixus* (Guárico, Venezuela) ♂	Absent	Absent	Absent	Absent	[27]
*R*. *robustus* (Trujillo, Venezuela) ♀ × *R*. *prolixus* (Guárico, Venezuela) ♂	Absent	Absent	Absent	Absent	[27]
*R*. *robustus* (Trujillo, Venezuela) ♀ × *R*. *prolixus* (Lara, Venezuela) ♂	Absent	Absent	Absent	Absent	[27]
*R*. *robustus* (Venezuela) ♀ × *R*. *prolixus* (Venezuela) ♂	Absent	Absent	Absent	Absent	[45]
*R*. *prolixus* (Cojedes, Venezuela) × *R*. *robustus* (Santander, Colombia)	Absent	Absent	Absent	Absent	[25, 26]
*R*. *prolixus* (Cojedes, Venezuela) × *R*. *robustus* (Lima, Peru)	Absent	Absent	Absent	Absent	[25, 26]
*R*. *prolixus* (Cojedes, Venezuela) ♀ × *R*. *robustus* (Pará, Brazil) ♂	Absent	Present^3^	–	–	[25, 26]
*R*. *prolixus* (Cojedes, Venezuela) ♀ × *R*. *robustus* (Rondônia, Brazil) ♂	Absent	–	–	–	[25, 26]
*R*. *prolixus* (Cojedes, Venezuela) × *R*. *prolixus*/*R*. robustus* (Boyaca, Colombia)	Absent	Absent	Absent	Absent	[25, 26]
*R*. *prolixus* (Lara, Venezuela) ♀ × *R*. *robustus* (Mérida, Venezuela) ♂	Absent	Absent	Absent	Absent	[27]
*R*. *prolixus* (Lara, Venezuela) ♀ × *R*. *robustus* (Trujillo, Venezuela) ♂	Absent	Absent	Absent	Absent	[27]
*R*. *prolixus* (Guárico, Venezuela) ♀ × *R*. *robustus* (Mérida, Venezuela) ♂	Absent	Absent	Absent	Absent	[27]
*R*. *prolixus* (Guárico, Venezuela) ♀ × *R*. *robustus* (Trujillo, Venezuela) ♂	Absent	Absent	Absent	Absent	[27]
*R*. *prolixus* (Venezuela) ♀ × *R*. *robustus* (Venezuela) ♂	Present	–	–	–	[45]
*R*. *prolixus* (Cundinamarca, Colombia) × *R*. *robustus* (Santander, Colombia)	Absent	Absent	Absent	Absent	[25, 26]
*R*. *prolixus* (Cundinamarca, Colombia) × *R*. *robustus* (Lima, Peru)	Absent	Absent	Absent	Absent	[25, 26]
*R*. *prolixus* (Cundinamarca, Colombia) ♀ × *R*. *robustus* (Pará, Brazil) ♂	Absent	Present^3^	Present (♀)^1,2^	–	[25, 26]
*R*. *prolixus* (Cundinamarca, Colombia) ♀ × *R*. *robustus* (Rondônia, Brazil) ♂	Absent	–	–	–	[25, 26]
*R*. *prolixus* (Cundinamarca, Colombia) × *R*. *prolixus*/*R*. robustus* (Boyaca, Colombia)	Absent	Absent	Absent	Absent	[25, 26]
*R*. *prolixus* (Honduras) × *R*. *robustus* (Santander, Colombia)	Absent	Absent	Absent	Absent	[25, 26]
*R*. *prolixus* (Honduras) × *R*. *robustus* (Lima, Peru)	Absent	Absent	Absent	Absent	[25, 26]
*R*. *prolixus* (Honduras) ♀ × *R*. *robustus* (Rondônia, Brazil) ♂	Absent	–	–	–	[25, 26]
*R*. *prolixus* (Honduras) ♀ × *R*. *robustus* (Pará, Brazil) ♂	Absent	–	Present (♀)^1,2^	–	[25, 26]
*R*. *prolixus* (Honduras) ♂ × *R*. *prolixus*/*R*. robustus* (Boyaca, Colombia) ♀	Absent	Absent	Absent	Absent	[25, 26]
*R*. *prolixus* (Honduras) ♀ × *R*. *prolixus*/*R*. robustus* (Boyaca, Colombia) ♂	Absent	Absent	–	Present (♂)^1,2^	[25, 26]
*R*. *prolixus* (Casanare, Colombia) × *R*. *robustus* (Santander, Colombia)	Absent	Absent	Absent	Absent	[25, 26]
*R*. *prolixus* (Casanare, Colombia) × *R*. *robustus* (Lima, Peru)	Absent	Absent	Absent	Absent	[25, 26]
*R*. *prolixus* (Casanare, Colombia) ♀ × *R*. *robustus* (Pará, Brazil) ♂	Absent	–	Present (♀)^1,2^	–	[25, 26]
*R*. *prolixus* (Casanare, Colombia) ♀ × *R*. *robustus* (Rondônia, Brazil) ♂	Absent	–	Present	–	[25, 26]
*R*. *prolixus* (Casanare, Colombia) × *R*. *prolixus*/*R*. robustus* (Boyaca, Colombia)	Absent	Absent	Absent	Absent	[25, 26]
*R*. *prolixus* (Amazonas, Brazil) ♀ × *R*. *robustus* (Pará, Brazil) ♂	Absent	–	Present (♂)^2^	–	[25, 26]
*R*. *prolixus* (Amazonas, Brazil) ♀ × *R*. *robustus* (Rondônia, Brazil) ♂	Absent	–	Present^1,3^	–	[25, 26]
*R*. *prolixus* (Amazonas, Brazil) ♀ × *R*. *robustus* (Santander, Colombia) ♂	Absent	–	Present^3^	–	[25, 26]
*R*. *prolixus* (Amazonas, Brazil) ♀ × *R*. *robustus* (Lima, Peru) ♂	Absent	–	Present (♂)^2^	–	[25, 26]
*R*. *prolixus* (Amazonas, Brazil) ♀ × *R*. *prolixus*/*R*. robustus* (Boyaca, Colombia) ♂	Absent	–	Present^1,3^	–	[25, 26]
*R*. *prolixus* (Amazonas, Brazil) ♂ × *R*. *prolixus*/*R*. robustus* (Boyaca, Colombia) ♀	Absent	Absent	Absent	Absent	[25, 26]
*R*. *prolixus*/*R*. robustus* (Boyaca, Colombia) × *R*. *robustus* (Santander, Colombia)	Absent	Absent	Absent	Absent	[25, 26]
*R*. *prolixus*/*R*. robustus* (Boyaca, Colombia) × *R*. *robustus* (Lima, Peru)	Absent	Absent	Absent	Absent	[25, 26]
*R*. *prolixus*/*R*. robustus* (Boyaca, Colombia) ♀ × *R*. *robustus* (Rondônia, Brazil) ♂	Absent	–	Present (♂)^2^	–	[25, 26]
*R*. *prolixus*/*R*. robustus* (Boyaca, Colombia) ♀ × *R*. *prolixus* (Amazonas, Brazil) ♂	Absent	Present^2^	–	–	[25, 26]
Intraspecific crosses					[25, 26]
*R*. *robustus* (Santander, Colombia) ♀ × *R*. *robustus* (Pará, Brazil) ♂	Absent	Present^3^	–	–	[25, 26]
*R*. *robustus* (Santander, Colombia) ♀ × *R*. *robustus* (Rondônia, Brazil) ♂	Absent	–	Present (♂)^2^	–	[25, 26]
*R*. *robustus* (Santander, Colombia) ♀ × *R*. *robustus* (Lima, Peru) ♂	Absent	Absent	–	Present (♀)^1, 2^	[25, 26]
*R*. *robustus* (Santander, Colombia) ♀ × *R*. *prolixus*/*R*. robustus* (Boyaca, Colombia) ♂	Absent	Absent	Absent	Absent	[25, 26]
*R*. *robustus* (Lima, Peru) ♀ × *R*. *robustus* (Pará, Brazil) ♂	Absent	–	Present (♀)^1, 2^	–	[25, 26]
*R*. *robustus* (Lima, Peru) ♀ × *R*. *robustus* (Rondônia, Brazil) ♂	Absent	–	–	–	[25, 26]
*R*. *robustus* (Lima, Peru) ♀ × *R*. *robustus* (Santander, Colombia) ♂	Absent	Absent	Absent	Absent	[25, 26]
*R*. *robustus* (Lima, Peru) ♀ × *R*. *prolixus*/*R*. robustus* (Boyaca, Colombia) ♂	Absent	Absent	Absent	Absent	[25, 26]
*R*. *robustus* (Pará, Brazil) ♀ × *R*. *robustus* (Santander, Colombia) ♂	Absent	–	–	Present^1,3^	[25, 26]
*R*. *robustus* (Pará, Brazil) ♀ × *R*. *robustus* (Lima, Peru) ♂	Absent	–	Present (♂)^1, 2^	–	[25, 26]
*R*. *robustus* (Pará, Brazil) ♀ × *R*. *robustus* (Rondônia, Brazil) ♂	Absent	–	–	Present^2,3^	[25, 26]
*R*. *robustus* (Pará, Brazil) ♀ × *R*. *prolixus*/*R*. robustus* (Boyaca, Colombia)	Absent	–	Present (♂)^1,2^	–	[25, 26]
*R*. *robustus* (Rondônia, Brazil) ♀ × *R*. *robustus* (Santander, Colombia) ♂	Absent	–	Present	–	[25, 26]
*R*. *robustus* (Rondônia, Brazil) ♀ × *R*. *robustus* (Lima, Peru) ♂	Absent	–	–	–	[25, 26]
*R*. *robustus* (Rondônia, Brazil) ♀ × *R*. *prolixus* (Pará, Brazil) ♂	Absent	–	Present^3^	–	[25, 26]
*R*. *robustus* (Rondônia, Brazil) ♀ × *R*. *prolixus*/*R*. robustus* (Boyaca, Colombia) ♂	Absent	–	Present (♂)^1,2^	–	[25, 26]

^*^R. *prolixus* according to collectors and *R*. *robustus* according to chromatic characters [24]; ^1^backcrosses; ^2^partial sterility; ^3^reduced fertility; ♀: female; ♂: male

## Discussion

Hybrids were obtained in all directions of the crosses (Table 1). However, crosses between *R*. *montenegrensis* females and *R*. *marabaensis* males resulted in a very low number of offspring (8%) (Table 1), suggesting extremely low interspecific fertility (possibly originating from one or more prezygotic barriers). Curiously, when we performed intercrossing, the few F1 hybrids showed high fertility (Table 1), possibly resulting from heterosis (or hybrid vigor). This phenomenon, previously suggested in *Triatoma* Laporte, 1832 hybrids [37], results in the phenotypic superiority of a hybrid over its parents with respect to traits such as growth rate, reproductive success and higher-yield [38].

In addition to F1, the F2 and the third-generation hybrids (F3) were fertile, being only the F4 unviable due to hybrid collapse (or hybrid breakdown) (Tables 1 and 2). This postzygotic barrier, previously characterized in Triatominae [32, 39], is related to the breakdown of the hybrid starting from F2 [24, 32]. It is believed that the breakdown (due to inviability and/or sterility) of these hybrids occurs due to genetic dysregulation [24, 32], that, including, result in chromosome pairing errors [32]—as demonstrated by cytogenetic analyses (Fig. 1A). These results highlight the need for experimental crosses up to at least F4 to correctly evaluate all possible postzygotic barriers, since if the reproductive compatibility analyses between *R*. *montenegrensis* females and *R*. *marabaensis* males had been closed in F1 or F2, the hybrid collapse would not have been characterized.

On the other hand, the hybrids of *R*. *montenegrensis* males and *R*. *marabaensis* females were completely infertile (Table 1), characterizing the hybrid sterility [21–24] (Table 2). Cytogenetic studies confirmed this reproductive barrier, since chromosome pairing errors were observed (Fig. 1B)—which result in nonviable gametes [40, 41] and, consequently, justify the absence of F2 hatching (Table 1). Barrett [25, 26] crossed populations of *R*. *robustus* from Brazil (Rondônia and Pará) that, given the current geographic distribution of the species [42], may represent *R*. *montenegrensis* and *R*. *marabaensis*, respectively, and also observed sterility and hybrid collapse [25, 26]. Hybrid sterility, together with prezygotic barriers, were the main mechanisms of reproductive isolation observed when *R*. *robustus s. l.* were crossed with *R*. *nasutus* and *R*. *neglectus* [25, 26] (Table 2).

As already mentioned, some authors consider that *R*. *robustus* II and *R*. *montenegrensis* are the same taxon [4, 5], although transcriptomic [17], morphological [15] and morphometric [15] studies highlight differences between these insects. Therefore, we performed crosses between *R*. *montenegrensis* and *R*. *robustus* II and hybrids were obtained in both directions up to F4 (Table 1). However, curiously, all F4 hybrid specimens from the cross between *R*. *montenegrensis* females and *R*. *robustus* II males died before reaching adulthood (characterizing the hybrid collapse [24]) (Table 2). Cytogenetic analysis of a N5 male nymphs demonstrated that these organisms presented pairing errors in one pair of autosomes (Fig. 1C). These findings are in accordance with the recent proposal by Filée et al. [2] who suggested that the clade *R*. *robustus* II is composed of *R*. *montenegrensis* and, possibly, another sister taxon of this species (or *R*. *prolixus* introgressed by mitochondrial DNA from *R*. *montenegrensis*).

*Rhodnius robustus* II was proposed on the basis of specimens from Brazil and Ecuador [3]. Subsequently, specimens from Bolivia were included in this clade [4]. As mentioned above, phylogenomic studies have shown that the *R*. *robustus* II clade is divided into two groups: one with specimens that were grouped with *R*. *montenegrensis* and the other with specimens of *R*. *robustus* II that do not represent *R*. *montenegrensis* (such as, for example, *R*. *robustus* II Lima, Peru originating from the same colony that we used in the crosses) [2]. Filé et al. [2] do not rule out the possibility that these specimens represent *R*. *prolixus* introgressed by mitochondrial DNA from *R*. *montenegrensis*. If we consider that *R*. *robustus* from Rondônia, Brazil used by Barrett [25, 26] represents *R*. *montenegrensis*, none of the crosses between this species and *R*. *prolixus* characterized hybrid collapse [only inviability and/or hybrid sterility, in addition to the absence of barriers in one or both directions of several attempts to cross with *R*. *prolixus* from Venezuela, Colombia, and Honduras—as observed in our crosses between *R*. *robustus* II females and *R*. *montenegrensis* males in which crosses were performed up to F5 and reproductive barriers (Tables 1 and 2) and chromosome pairing errors (Fig. 1D) were not observed] [25, 26], highlighting the need for further studies with the *R*. *robustus* II clade (mainly with field material) to try to elucidate this taxonomic problem.

When males *R*. *robustus* from Lima, Peru were crossed with females of *R*. *robustus* from Santander, Colombia, hybrid collapse was also characterized [25, 26] (Table 2). However, when Barrett [25, 26] performed crosses similar to those presented in Table 1, namely, *R*. *robustus* from Lima, Peru with *R*. *robustus* from Rondônia, Brazil (which could represent *R*. *montenegrensis* by geographic distribution [42]), no reproductive barrier was observed by the author (Table 2). It is important to emphasize that the author only performed interspecific crosses, intercrossing and backcrossing [25, 26], which would not allow characterizing the hybrid breakdown present only in F4 (Table 1). Furthermore, on the basis of the notification of *R*. *montenegrensis* in other South American countries (Bolivia [43] and, more recently, Peru [44]) and on the description of *R*. *barretti* Abad-Franch et al. [45] (from Colombia) as a related species to *R*. *robustus s. l.* [45], we cannot rule out the possibility that *R*. *robustus* from Peru and Colombia used by Barrett [25, 26] could be *R*. *montenegrensis* and *R*. *barretti*, respectively.

As already mentioned, many crossings between *R*. *robustus s. l.* (Colombia, Peru and Venezuela) and *R*. *prolixus* (Honduras, Colombia and Venezuela) did not present reproductive barriers [25–27]. Among all the crossing attempts, only two presented reproductive barriers: females *R*. *prolixus* from Venezuela with males *R*. *robustus* from Venezuela (prezygotic barrier) [46] and females *R*. *robustus* from Santander, Colombia with males *R*. *robustus* from Lima, Peru (hybrid collapse) [25, 26] (Table 2). However, most crosses between *R*. *prolixus* (all localities) with *R*. *robustus* from Brazil presented postzygotic barriers (Table 2): practically all crosses with *R*. *robustus* from Pará (possibly *R*. *marabaensis*), for example, presented hybrid sterility (Table 2). Furthermore, all crossing attempts between *R*. *prolixus* from Amazonas, Brazil, and *R*. *robustus s. l.* resulted in reproductive barriers (Table 2). There is a question about the presence of *R*. *prolixus* in Brazil [31, 42]. If this is confirmed, the reproductive barriers observed by Barrett [25, 26] indicate that this taxon is not *R*. *robustus*, *R*. *montenegrensis*, and *R*. *marabaensis* (since intraspecific crosses cannot result in reproductive isolation [21]).

## Conclusions

We demonstrated that, on the basis of the biological species concept, *R*. *montenegrensis* and *R*. *marabaensis* are valid species and reproductively isolated. Furthermore, we observed partial isolation between *R*. *montenegrensis* and *R*. *robustus* II, highlighting the need for integrative taxonomy studies (with field insects) to elucidate this clade. Finally, we revisited the literature and observed that *R*. *robustus s. l.* is reproductively isolated from *R*. *nasutus* and *R*. *neglectus*, we emphasize that, with the exception of *R*. *prolixus* from Amazonas, most other allopatric populations of *R*. *prolixus* did not show reproductive isolation when crossed with *R*. *robustus* (from Colombia, Peru, and Venezuela) and we discuss the “intraspecific” barriers of *R*. *robustus s. l.* (which possibly represent specimens of *R*. *montenegrensis*, *R*. *marabaensis*, and/or *R*. *barretti*).

## Data Availability

Data are provided within the manuscript or supplementary information files.
